# Benefits of Reflective Writing in Health Care through the Vivid Lens of House Officers

**DOI:** 10.15694/mep.2020.000060.1

**Published:** 2020-03-31

**Authors:** Anbreen Aziz, Usman Mahboob, Tayyaba Saleem

**Affiliations:** 1 Hazrat Bari Sarkar Dental (HBS) College; 2 Khyber Medical University (KMU); 3 Dental Section

**Keywords:** Reflective writing, critical thinking, self-regulation, clinical behavior.

## Abstract

This article was migrated. The article was marked as recommended.

**Introduction:** Reflective writing, a complex human activity is one of the innovative pedagogies to promote deep learning among medical students and doctors. Despite its potential to facilitate learning, there is limited literature on evaluation of various purposes of reflective writing in medical education. Hence, aim of this study is to develop an instrument and evaluate the perceptions of house officers about benefits of reflective writing.

**Methods**:Mixed method study followed AMEE 87 guidelines for questionnaire development. The study was carried out from Oct 2018-Feb 2019 in a dental college in Islamabad. A 30-items questionnaire was developed by following these steps: (1) conduction of literature review, (2) item development, (3) conduction of cognitive interviews and (4) pilot testing. Coding and interpretation of transcribed data and notes taken during cognitive interviews was done to finalize three main themes (learning, self-regulation and alteration in clinical behavior) identified in literature review. In pilot testing, participants were asked to rate the purposes of reflective writing on a three-point Likert scale (Agree, do not know and disagree). Data was analyzed using SPSS version 22.

**Results:** All of nineteen house officers (n= 3 for cognitive interviews, n= 16 for pilot testing) had previous experience of writing reflections using Gibb’s reflective cycle. Thirteen (81%) out of sixteen house officers agreed that reflective writing improves learning, helps in self-regulation and alters clinical behavior, two (13%)did not know about the three themes that were finalized in cognitive interviews and one (6%) did not agree.

**Discussion and Conclusion:**Reflective writing improves learning, helps in self-regulation and alters clinical behavior in the selected house officers. This study may inspire medical education experts to include reflective writing as a part of formal undergraduate medical and dental curriculum to enhance student’s learning experience.

## Introduction

Medical education help students to acquire knowledge, skills and attitudes necessary for the medical health profession ( Schei, Fuks and Boudreau, 2018). Therefore, it is the responsibility of medical teachers to introduce right pedagogy among their students ( Sukhato *et al.*, 2016). Reflective writing is one of the latest pedagogies being used in medical education. It is a complex human activity ( Anne de la Croix, 2018). It has been proliferating in educational programs to enhance development of reflective capacity, to extend empathy by deep understanding of patient’s experiences of their illness and to promote practitioner well-being ( Boud and Walker, 1998; Wald and Reis, 2010). Hence, the ultimate goal of medical education is to produce knowledgeable, up to date and skillful professionals with undertaking of maintaining and developing expertise over the period of their lifelong career ( Swanwick, 2014).

Reflection is a metacognitive process in which a person is engaged in attentive, critical, exploratory and iterative interactions with his thoughts and actions to change them, hence it is powerful equipment of experiential learning ( Fragkos, 2016; Larsen, London and Emke, 2016; Asiah Mohd Sharif, 2017). Attention to self and critical reflection on the situation are two necessary elements for continued competence (Sanders, 2015; Fragkos, 2016). Reflective writing engages one in the process of deep understanding and continuous learning ( Jorwekar, 2017). It can improve individual’s specific learning situation to have greater self-awareness, professional expertise, critical thinking and resilience, therefore provide improved service to clients ( Jorwekar, 2017). Active thinking is only possible when we reflect upon events about what we have done good or bad, how differently we have done it and how it can be done in a better way ( Chesterman, 2014). However, ‘reflective zombie’ is the term used in the literature for those students who just follow the steps of the thought process they are being told rather to engage themselves in authentic reflection process ( Anne de la Croix, 2018).

Several frameworks have been developed and are used for reflective practice to enhance student’s learning experience and professional development. John Dewey in 1938, Schon in 1983, Kolb in 1984 and Driscoll in 1994 introduced various models for reflection ( Donald A. Schon, 1984). Whereas the most useful framework is given by Gibbs in 1988 and he introduced a *structured reflective cycle* consisting of organised stages ( Gibbs, 1988; Priddis and Rogers, 2017).

Reflective practice in teaching is used as a self-assessment tool. It is also useful for professional development, enhancing student’s learning experience and improving memory as it is used to recall previous experience ( Larsen, London and Emke, 2016). Usually reflective diaries, portfolios, blogs, journals, poetry, some short stories, novels or books are used for written reflections ( Korthagen, 1993). Most of the work on reflective writing has been done in nursing ( Moattari, 2007) and Larsen *et al*. (2016) stated that it is rarely a formal part of the daily work of medical education or practice. Checklists, portfolios and other tools that are being used to encourage reflection that are isolated from original theories of reflection and reflective practice, hence the main essence of reflection has been lost ( Larsen, London and Emke, 2016). Despite the importance of reflective writing, there is general lack of awareness of different purposes of reflection for learning. Literature search revealed a gap that there is a need of in-depth inquiry regarding reflection. Hence, there is a need to develop an instrument that can evaluate various purposes of reflective writing ( Mann, 1999; Mann *et al.*, 2007; Fragkos, 2016).

## Methods

Mix method study with sequential qualitative and quantitative components following guidelines of AMEE 87 for questionnaire development in educational research was carried out over five months (Oct 2018-Feb 2019) in one of the dental colleges in Islamabad. Selected house officers had previous experience of reflective writing during their rotation in the Prosthodontic department.

***Data Collection:*** Approval of the study was obtained from Institutional Review Board Committee of the institute. A purposive sampling (n=19) of house officers was done for cognitive interviews and later pilot testing. The participants selection was based on their written reflections according to Gibb’s Reflective Cycle during their routine rotation in the Prosthodontic department (reflective writing is compulsory part of their logbook in Prosthodontics rotation only). For cognitive interviews, three categories of house officers were selected (average, above average and below average) based on their undergraduate academic record of final professional examination provided by the administration upon request. The purpose of choosing three categories of house officers was to get maximum input of their responses to finalize items. Participants were informed about the research implication and their participation was voluntary. Informed consent was taken before cognitive interviews and the pilot testing. House officers who participated in cognitive interviews were excluded in pilot testing.

***Questionnaire*** ***:*** A 30-items questionnaire (Supplementary File 1) was developed by following these steps: (1) conduction of literature review, (2) item development, (3) conduction of cognitive interviews from three house officers and (4) pilot testing. The initial questionnaire had 32 items under four themes emerged from literature review (learning, self-regulation, alteration in clinical behavior and organizational skills) which were then reduced to 30-items under three themes after cognitive interviews. The first theme was “learning” which comprised of seven items. The second theme “self-regulation” included fourteen items and the third theme was “alteration in clinical behavior” that comprised of nine items.

Three-point Likert scale (agree, do not know and disagree) was used to ask participants to rate the purposes of reflective writing. The rationale behind using three-point Likert scale was to get clear responses. House officers at early career may have been unable to distinguish between the narrow boundary of strongly agree and agree or vice versa. This was evaluated in the cognitive interviews and during discussion about the points of Likert scale.

***Data collection technique for cognitive interviews and pilot testing:*** After an informed consent and appropriate briefing, data was first collected from cognitive interviews from three selected house officers for quality assurance procedure. The technique used was a mixture of think aloud and concurrent verbal probing as it better identifies potential errors ( Artino *et al.*, 2014). A newly developed questionnaire was then distributed among sixteen house officers for pilot testing.

***Data Analysis:*** For cognitive interviews,coding and interpretation of transcribed data and written notes taken during the interview was done for alterations in the items. For pilot testing, the data was analyzed using SPSS version 22. Frequencies were calculated for all items as well as the items under three themes (learning, self-regulation and alteration in clinical behavior).

Ethics approval for this study was granted on 20th November 2018 by Dr. Hina Mahmood, Secretary, Institutional Review Board Committee of Dental Section, Islamabad Medical and Dental College, Islamabad (Ref IMDC/DS/OG/280).

## Results/Analysis

Among nineteen participants of the study, three participated in cognitive interviews and remaining sixteen participated in pilot testing (100% response rate). See Table 1:

**Table 1.** Participant’s demographics

No of participants	Average Age (Years)		Gender	
Cognitive interviews	03	23	01 M	02 F
Pilot testing	16	23	06 M	10 F

Cognitive Interviews:

The decision of rephrasing, omitting and repositioning the statements of few items was done after conduction of cognitive interviews. See Table 2:

**Table 2.** Results of cognitive Interviews from three participants

Item No (initial/ final)	Item’s statement before conducting cognitive interviews	Categorical response by participants	Coding and interpretation by author	Finalized items for pilot testing (Total=30)
**2/ 2**	Do you think reflection re-organizes your previous knowledge for **real life?**	Above Average Elaborate real life	Rephrase	Practical life
**4/ 4**	Do you think reflection has marked effect (impact) on self-directed and **continuous learning** with aim of improving knowledge skills and competences **for personal reasons or to develop career skills?**	Below Average Change continuous learning to ongoing learning or lifelong learning	Rephrase	Lifelong learning Personal and professional growth?
**6/ 6**	Do you think reflection helps in **improving** understanding of self and **situation?**	Above Average Explain situation and re-write complete sentence	Rephrase	Developing Present situation so that future encounters with the similar situation are informed from the previous encounters
**7/ 7**	Do you think reflection is an ongoing process and its **value** depends upon **repeated cycles of action, reflection and action?**	Average Confusing and time taking statement. Make it clear Below Average Not clear about the action, reflection and again reflection	Rephrase	Importance Repeated cycles of any action, then reflecting on it and then again doing modified action
**9/ 9**	Do you think reflection enhances quality of your work to achieve your **goals?**	Above Average Explain which goals?	Rephrase	Personal and professional goals
**11/ 11**	Do you think reflection is a process with a definite purpose that changes your **future response to situations?**	Above Average Make it future response to similar situation Average Dealing situation next time? Below Average Future response is not clear	Rephrase	Modifies your future response to similar situation.
**12/ 12**	Do you think reflection **before an action** has the potential for greater personal growth and learning?	Below Average Make statement easy regarding reflection before action	Rephrase	Before doing any action/ procedure
**14/ 14**	Do you think deeper reflections possibly change your **beliefs and point of view?**	Above Average Change beliefs into existing beliefs or just write point of view	Rephrase	Point of view about anything
**17**	Do you think reflection helps in documentation?	Above Average and Average Same question as filing and record keeping	Omit	Omitted
**19/ 23**	Do you think reflection helps in teamwork?	Above Average More related to theme alteration in clinical behavior	Re-position	Re-positioned at no 23 under theme alteration in clinical behavior
**24/ 22**	Do you think reflection enables you to improve your **ability to work with patients?**	Below Average Make it desire to work with patients	Rephrase & Re-position	Desire to work with patientsRe-positioned at no 22
**25**	Do you think reflection helps you to work with colleagues in a professional manner?	Above average Already included as teamwork	Omit	Omitted
**26/ 24**	Do you think reflection makes your **communication** purposeful?	Below Average Is it about communication skills?	Rephrase & Re-positioned	Improves your communication skillsRe-positioned at no 24
**28/ 26**	Do you think reflection increases your general confidence **to interact with patients?**	Above Average Add colleagues along with patients	Rephrase & Re-position	Interact with patients and colleagues Re-positioned at no 26
**30/ 28**	Do you think reflection helps in reducing **medical errors?**	Below Average Medical errors are not clear Average Related to medical or dental?	Rephrase & Re-position	Treatment errors Re-positioned at no 28
**32/ 30**	Do you think reflection increases problem solving ability of **complex and unusual cases?**	Average Explain complex and unusual cases	Rephrase & Re-position	Complicated cases Re-positioned at no 30

Pilot Testing:

Thirteen **(81%)** out of sixteenrespondent house officers agreed that reflective writing improves learning, helps in self-regulation and alters clinical behavior. Two **(13%)** did not know about the three themes that were finalized in cognitive interviews and one **(6%)** did not agree. Sixteen house officers **(100%)** agreed that reflection helps in understanding self and situation, monitoring own work progress, setting goals and meeting them in time. See Table 3:

**Table 3.** Frequencies and percentages of three themes originated from literature review and confirmed in cognitive interviews

THEME 1: LEARNING							
	AGREE	DO NOT KNOW			DIS-AGREE		
FREQUENCY (Number of participants; **F**)	14	1			1		
PERCENTAGE (%, **P**)	87.5	6.2			6.2		
**ITEM NO**	**ITEM CODE**	**F**	**P**	**F**	**P**	**F**	**P**
**1**	Depth of knowledge	12	75	4	25	none	none
**2**	Re-organization of previous knowledge	15	93.8	1	6.3	none	none
**3**	Improvement in procedural skills	12	75	2	12.5	2	12.5
**4**	Self-directed and lifelong learning	14	87.5	2	12.5	none	none
**5**	Responsibility for own learning	15	93.8	1	6.3	none	none
**6**	Understanding of self and present situation	16	100	none	none	none	none
**7**	Ongoing process with modified action	14	87.5	2	12.5	none	none
**THEME 2: SELF-REGULATION**							
	**AGREE**	**DO NOT KNOW**			**DIS-AGREE**		
FREQUENCY (Number of participants, **F**)	11	3			2		
PERCENTAGE (%, **P**)	68.7	18.7			12.5		
**ITEM NO**	**ITEM CODE**	**F**	**P**	**F**	**P**	**F**	**P**
**8**	Monitoring own work progress	16	100	none	none	none	none
**9**	Enhancement in work quality	13	81.3	2	12.5	1	6.3
**10**	Thinking about strengths and weaknesses	14	87.5	2	12.5	none	none
**11**	Modification in future response	14	87.5	2	12.5	none	none
**12**	Greater personal growth and learning	14	87.5	2	12.5	none	none
**13**	Improvement in outcome of situation	13	81.3	2	12.5	1	6.3
**14**	Change point of view by deeper reflection	10	62.5	5	31.3	1	6.3
**15**	Self-management	12	75	2	12.5	2	12.5
**16**	Management of schedules	6	37.5	4	25	6	37.5
**17**	Record keeping	6	37.5	6	37.5	4	25
**18**	Multitasking	7	43.8	6	37.5	3	18.8
**19**	Goal setting and meeting them in time	16	100	none	none	none	none
**20**	Decision making	9	56.3	3	18.8	4	25
**21**	Resolution of serious disagreement at workplace	8	50	7	43.8	1	6.3
**THEME 3: ALTERATION IN CLINICAL BEHAVIOR**							
	**AGREE**	**DO NOT KNOW**			**DIS-AGREE**		
FREQUENCY (Number of participants, **F**)	13	2			1		
PERCENTAGE (%, **P**)	81.3	12.5			6.3		
**ITEM NO**	**ITEM CODE**	**F**	**P**	**F**	**P**	**F**	**P**
**22**	Improvement in desire for clinical work	11	68.7	2	12.5	3	18.8
**23**	Teamwork	13	81.3	3	18.8	none	none
**24**	Improvement in communication skills	12	75	1	6.3	3	18.8
**25**	Increased self-confidence	15	93.8	1	6.3	none	none
**26**	Increased general confidence	13	81.3	3	18.8	none	none
**27**	Increased desire for more theoretical knowledge and learning new skills	13	81.3	3	18.8	none	none
**28**	Reduction in treatment errors	13	81.3	1	6.3	2	12.5
**29**	Better patient care	12	75	3	18.8	1	6.3
**30**	Increased problem-solving ability	12	75	3	18.8	1	6.3

## Discussion

The present study has evaluated the perceptions of house officers about the various purposes of reflective writing. Majority of the house officers (13/ 16; 81%) in pilot testing agreed that reflective writing does enhance learning, helps in self-regulation and alters clinical behavior.

Frenk *et al*. (2010) stated that outdated and static curricula without addition of innovative methods for students learning is the reason for production of ill-equipped graduates from underfinanced institutions as it mismatches professional competencies, patient and population priorities ( Julio Frenk, Lincoln Chen, Zulfiqar A Bhutta, Jordan Cohen, Nigel Crisp, 2010). In medical education, various learning theories have been provided in literature and reflective practice in health care education is one of those learning theories ( Dacre, 2001). Mostly reflections have been explored in nursing and limited data is available for the reflective writing in medical and dental schools ( Moattari, 2007). Previously, it has been explored that writing reflections every day for two consecutive weeks had positive learning influence on medical students analyzing their clinical performance ( Larsen, London and Emke, 2016).

In our study fourteen (88%) house officers agreed that reflection improves learning. It does improve learning due to critical thinking ( Jorwekar, 2017). A systematic review stated that reflective writing is related to learning, professional identity development, and critical thinking in medical and health professions students ( Fragkos, 2016). Thirteen house officers (81%) agreed that reflection increases desire for more theoretical knowledge and learning new practical skills. Personal knowledge incorporates knowledge of skills and practices and personal understandings of people and situations ( Michael Eraut, 2010). Nine studies in a systematic review reported that reflections in clinical portfolios helped in measurable change in student skills and attitudes and one study reported a change in student behavior ( Buckley *et al.*, 2009).

Reflective practice leads to deeper understanding from experience or situation and enable us to understand our strengths, acquisition of knowledge, skills, attitudes and values ( Fragkos, 2016). In our study, fourteen (88%) house officers agreed that reflection enables one to think about one’s strengths and weaknesses. While doing clinical work one may think of the strengths and weaknesses of the particular situation and for the next time those weaknesses can be managed for the better output. Comparing with our study, sixteen (100%) house officers agreed that reflection helps in developing understanding of the self and present situation so that future encounters with the similar situation are informed from the previous encounters. Hence, another important aspect of reflective practice is enhancement of patient care for the health professionals ( Fragkos, 2016). We also had similar finding in our study where twelve (75%) house officers agreed that reflection helps in better care of patients.

Reflection also leads to motivation and self-directed learning ( Fragkos, 2016). Reflective practice maximizes deep and life-long learning ( Hargreaves, 2016). In our study fourteen (88%) participants agreed that reflection has marked effect on self-directed and lifelong learning and twelve (75%) agreed that it improves depth of knowledge. Jorwekar (2017) stated that self-directed learning is the important aspect of adult learning in medical education. Reflection not only increases skill and knowledge but also helps students in communication. Reflection is the strategy by which attitude and communication among students can be inculcated. Present medical education needs to include such methods which can assure strong communication skills ( Jorwekar, 2017). Hence communication between health care professional and patient is very important. In our study twelve (75%) agreed that reflection improves communication skills. One more advantage of reflection is that it also changes student’s behavior ( Fragkos, 2016). If one reflects on the clinical experience, one can reduce medical errors by changing clinical behavior, learning new knowledge and practical skills ( Fragkos, 2016). In our study thirteen (81%) agreed that reflection alters clinical behavior and it reduces treatment errors.

Reflective writing strategy if used regularly and effectively with supervision, can have various roles and benefits among doctors and undergraduate medical students. It may help them in becoming better future health care professionals by inculcating the habit of life-long learning ( Taranikanti *et al.*, 2019). Limited studies are available in the literature that have evaluated the main purposes of reflective writing in depth. Reflective writing shall be included in the undergraduate curriculum from the initial phase of medical school due to its vast benefits as shown in our study.

***Limitations:*** We only used four out of seven steps to develop questionnaire as it served the purpose of our study ( Artino *et al.*, 2014). We obtained only the response process validity from potential respondents through cognitive interviews. Moreover, the study could have been expanded to include multiple institutes with large sample size and to compare the differences in different cohorts of house officers working in different institutional settings.

## Conclusion

This study confirms that reflective writing enhances learning, helps in self-regulation and alters clinical behavior of dental house officers in the preliminary findings from pilot testing. The results of this study may inspire medical educationists in Pakistan to include reflective writing as a part of formal undergraduate and postgraduate medical and dental curriculum to develop the habit of reflection and to enhance student’s learning experience so that they may gain more knowledge and improve their practice.

## Take Home Messages

• Reflective writing should be made mandatory for dental students going through clinical clerkships and house officers as this will help to inculcate a habit of reflective learning for the future professional life.
• The importance and purpose of reflective writing needs to be emphasized from the beginning of medical education.

## Notes On Contributors

**Anbreen Aziz** is a Dental Health Professional in the Department of Medical Education at Hazrat Bari Sarkar (HBS) Dental College, Islamabad, Pakistan.

**Usman Mahboob** is Director of Institute of Health Professions Education & Research (IHPER) at Khyber Medical University (KMU) Peshawar, Pakistan.

**Tayyaba Saleem** is a Clinical Professor and Head of Department of Prosthodontics and Dental Health Professional in the Deprtment of Medical Education at Dental section of Islamabad Medical and Dental College (IMDC), Islamabad, Pakistan.
