# Supplementary material for: Benefits of Reflective Writing in Health Care through the Vivid Lens of House Officers
Source: MedEdPublish (2016). 2020 Mar 31;9:60. [Version 1] doi: 10.15694/mep.2020.000060.1 (PMC10697461; doi:10.15694/mep.2020.000060.1)
Supplement: Peer review history

# Reviewer report: Reviewer response for version 1

**DOI:** 10.21956/mep.19986.r30411

**Published:** 2020-04-29

**Authors:** Samar Ahmed

**Affiliations:** 1 Ain Shams University Faculty of Medicine

This review has been migrated. The reviewer awarded 4 stars out of 5 What a beautiful piece of research. Reflective writing and its study through the perception of house officer learners is an outstandibg topic. The topic is by Far the deepest technique to tackle deep learning. I am particularly happy with the methodology an adsition might be called for for a longitudinal study on these learners to establish achievement and attainment .

Reviewer Expertise:

NA

No decision status is available

# Reviewer report: Reviewer response for version 1

**DOI:** 10.21956/mep.19986.r30410

**Published:** 2020-04-08

**Authors:** BALAJI ARUMUGAM

**Affiliations:** 1 TAGORE MEDICAL COLLEGE AND HOSPITAL

This review has been migrated. The reviewer awarded 5 stars out of 5 THANKS FOR THE INVITE TO REVIEW THIS ARTICLE. The research article adapted mixed method study design which is a commendable work and the methodology, scientific approach, qualitative analysis, expression of the results were clear and kudos to the entire team of authors for doing a fantastic work on the usually non practiced but recommended by the medical educators worldwide. The study concludes that reflective writing enhances learning, helps in self-regulation and alters clinical behavior of dental house officers. My questions to the authors: 1. Is this writing the reflection daily / weekly / regular activity among dental house officers?2. Did the participants had any formal training / workshops in medical education to write reflection? 3. I got fascinated with the qualitative results of cognitive interviews - its surprising that the dental house officer not even having post graduate degree how come they were able to give so many responses clearly (Close to final responses)??? - Please clarify????

Reviewer Expertise:

NA

No decision status is available

# Reviewer report: Reviewer response for version 1

**DOI:** 10.21956/mep.19986.r30412

**Published:** 2020-04-07

**Authors:** Mohamed Al-Eraky

**Affiliations:** 1 University of Dammam

This review has been migrated. The reviewer awarded 5 stars out of 5 Thank you for sharing this interesting study. The authors reported the perceptions of house officers on the significance of reflective writing on their learning, self-regulation and alteration of clinical behaviors. The manuscript is well-organized, and the discussion is coherent and structured. Good job! Participants of the study reported that reflection alters clinical behavior and it reduces treatment errors. This point may be a venue for a future study to investigate ‘how’ reflective writing actually contribute to improved learning and better clinical practice using examples from their clinical practice. Yet, perhaps instead of house-officers, dentists might be recruited to provide evidence on how reflection advances their practice. Another point related to the application of reflection models. As you know, there’s no such thing as one model fits all in medical education. For instance, ‘each’ institutional design model, (e.g. Gagne, Merrill, ADDIE, 4C/ID, …etc.) can be used for particular types of session, based on different contextual variables. Likewise, it would be interesting for the authors plan a future study on how and why to select the appropriate framework for reflection (e.g. Gibbs, Kolb, Schön, Driscol, …etc.). This would provide a valuable hands-on manual for reflection. I hope my comments would motivate you to plan a series of future studies, inspired by this one. Good luck!

Reviewer Expertise:

NA

No decision status is available

# Reviewer report: Reviewer response for version 1

**DOI:** 10.21956/mep.19986.r30408

**Published:** 2020-04-03

**Authors:** Sateesh Babu Arja

**Affiliations:** 1 Avalon University School of Medicine

This review has been migrated. The reviewer awarded 4 stars out of 5 It is a very interesting manuscript. I read it with much interest. Reflective writing became an integral part of the medical education, especially in workplace-based settings. Reflective writing in medical education is followed in many ways, including logbooks or work diaries or portfolios. The literature review in this article is up to date, and references are relevant. But I am a little bit confused with the research methodology. I have read the previous reviewers' comments and authors' replies. I am not aware of AMEE's guide for the development of the questionnaire. If I were the author, I would have called the first three interviews as pilot testing for developing questionnaire, and other 16 interviews are as part of the actual study (Just a thought). The other comment which is a surprise time that there was no mention of Kolb's reflective learning theory in the entire manuscript. The discussion has drawn appropriately based on results. Thank you.

Reviewer Expertise:

NA

No decision status is available

# Reviewer report: Reviewer response for version 1

**DOI:** 10.21956/mep.19986.r30406

**Published:** 2020-04-01

**Authors:** Deb Halder

This review has been migrated. The reviewer awarded 5 stars out of 5 Note on Title, Abstract and ReferencesThe title of this paper is well articulated that provides what the paper tends to explore and establish. The abstract serves all the required information of the paper so that the paper can be visualized. The references are up to date with recent findings of researchers though few have been used from 80s and 90s publications but not yet irrelevant.Note on Introduction and Methodology:The introduction of this paper is evidence based, orderly and argumentative. But the authors could have provided a research question that fosters a hypothesis and this could have provided a directed objective without its tacit expression of all these.The methodology provided detail of the way and procedures of the research which is imitable, adaptable for any researcher from any corner of the world.Note on Results and Discussion: The results and discussions of this paper is vivid and clear. The strength of this paper is to have triangulated the quantitative and qualitative study. The table is vivid and there is no discrepancy of interpretation with the scope of the paper. The authors could have added few suitable graphs in order to make the results more readable. Conclusion:The authors have firm conviction on the limitation of the research which is praiseworthy and honest approach. The concluding remark is very knit and condensed that explains the results and discussions in a nut shell. Thanks for the effort for gifting such a well-constructed paper.

Reviewer Expertise:

NA

No decision status is available

# Reviewer report: Reviewer response for version 1

**DOI:** 10.21956/mep.19986.r30407

**Published:** 2020-03-31

**Authors:** P Ravi Shankar

**Affiliations:** 1 American International Medical University

This review has been migrated. The reviewer awarded 4 stars out of 5 This is an interesting article on reflective writing and its use in health professions education. Reflective writing is being increasingly used though as the authors mention it may not always be used correctly. I was not aware of the term ‘cognitive interviewing’. The authors can explain this in detail for the benefit of the readers. Gibb’s reflective cycle can also be explained briefly. I assume there were two main objectives for this paper. The first was to examine the evidence in favor of reflective writing in health professions education. The second was to develop a questionnaire through review of literature and the process of cognitive interviewing and pretest the same. The authors can reframe the manuscript so that these two objectives are kept separate in my opinion.A description of how exactly the process of cognitive interviewing was used to develop the questionnaire can be provided. Tables 2 and 3 do describe this process but may need greater clarification for readers. What were the parameters examined during pre-testing of the questionnaire? Did this result in any modification of the instrument? Are three respondents enough for the purpose of cognitive interview? The questionnaire developed by the authors can be shown in the Appendix. Some reorganization may be required to further strengthen the flow and development of ideas.

Reviewer Expertise:

NA

No decision status is available

# Reviewer report: Reviewer response for version 1

**DOI:** 10.21956/mep.19986.r30409

**Published:** 2020-03-31

**Authors:** Felix Silwimba

**Affiliations:** 1 University of Lusaka

This review has been migrated. The reviewer awarded 5 stars out of 5 this article informs us of a very important dimension to medical education. reflective learning is important for our generation of learners and should be encouraged everywhere. I'm motivated and encouraged by this article.

Reviewer Expertise:

NA

No decision status is available
